# Axial compressor blades tip leakage flow control using natural aspiration

**DOI:** 10.1038/s41598-023-49391-9

**Published:** 2023-12-19

**Authors:** Peyman Ghashghaie Nejad, Reza Taghavi Zenouz

**Affiliations:** School of Mechanical Engineering, Iran University of Science and Technology (IUST), Narmak, Tehran, 16846-13114 Iran

**Keywords:** Energy science and technology, Engineering

## Abstract

Blades tip leakage flow structure in axial compressors has dominant effects on flow stability and losses. Accordingly, in the present study, three different ideas are introduced for alleviation of undesirable effects of the blades tip leakage flow. These ideas utilize circumferential tape, S-shape nozzle and natural aspiration slot, all imposed upstream the rotor blades row. The method of investigation is based on numerical simulation of the governing flow field. Results, in terms of the flow field structure and performance curves are compared with those of the untreated case. Final results, showed that the natural aspiration idea works better than the other ones. In comparison to the plane case, it is accompanied by augmentation of 3.5% in the total pressure ratio and 3% in the surge margin. It was also found that the mass flow rate passing through the blades row tip gap has increased by 2.33 g/s in the natural aspiration case.

## Introduction

Compressor unit is a key component of a gas turbine engine. Inherent flow leakage at the rotor blades tip gap region can cause considerable losses in the total energy. In addition, the tip leakage vortex flow may impose significant blockage to the main flow in the blades tip region, and as a result, can magnify the flow instabilities.

Blades tip leakage flow is basically due to the combination of the mainstream and the flow caused by the pressure difference between either sides of the blade at its tip region. Requirement of high thrust to weight ratio in modern aero-engines encourages designers to consider high loading per each stage of the compressor unit. This makes the blade tip vortex flow and the consequent blockage to the main flow at this region to strengthen. Consequently, the losses increase which degrades the compressor aerodynamic performance. Generally speaking, the tip leakage flow losses may constitute about 20–30% of the total losses^1^.

Of pioneers who have studied on the tip leakage flow in turbomachines can be referred to Rains^2^. He had used various theoretical models to simplify the flow mechanism and mixing process of the tip leakage flow^2,3^. So far, many attempts are made to numerically simulate the rotor blades tip vortical flows. Phillips et al.^4^ examined the end-wall boundary layer within an axial compressor while it passes through its rotating blades. They found out that the trailing vortex sheet originated from the blade tip are swamped and rapidly dispersed by the large-scale motions in the turbulent end-wall layer. Zhang et al.^5^ have analyzed unsteady flow characteristics and fluctuation mechanism in a transonic compressor rotor at near stall condition. They found that at under this condition, strong unsteady fluctuations appear on the blade pressure surface near its leading edge region downstream the shock waves. Li et al.^6^ through their casing pressure measurements and stereoscopic particle-image velocimetry (PIV) characterized behavior of the rotor tip leakage flow at both the design and near-stall conditions in a low-speed multistage axial compressor.

Up to now, many attempts are made to increase the compressor operating range using active and passive control methods. However, casing treatments of passive type in axial compressors are among the simplest and cheapest methods for controlling the flow instabilities. In addition, they have less impact on the engine total weight and the compressor efficiency^7^.

Proper casing treatment can enhance the tip leakage flow characteristics. This regional beneficial effect can extend towards the blade root region, and as a result, can improve the aerodynamic performance of the blade, nearly all along its span. Taghavi et al.^8^ experimentally controlled the blades tip leakage flow in a low speed axial compressor by applying very low rate of air injection via 12 nozzles mounted evenly spaced around the casing circumference. They observed, through smoke and tuft flow visualizations, that the beneficial effects of proper air injection at the blades tip region remarkably extends nearly along the whole blade span. Their hot-wire anemometry attempts supported the above conclusion, too.

A low-speed axial compressor with casing treatment of axial slots type was numerically examined by Hwang and Kang^9^. They showed that removal or injection of flow through the axial slots are responsible for extension of the operating range and alleviation of the unsteadiness. Their analyses of instantaneous flow field properties clarified the mechanism of the interaction between the treated casing and the unsteady oscillation of the tip leakage flow. They also evaluated impacts of different re-circulation rates and location of removal or injection of air flow on the unsteadiness of the tip leakage flow.

In this research work, three new methods are proposed to control or improve the blades tip vortical flow. These methods include using circumferential tape, S-shape nozzle and natural aspiration slot all imposed upstream the rotor blades row. The method of investigation is based on numerical calculations. Flow field for each case is analyzed quantitatively and qualitatively. Finally, the best casing treatment case is identified and introduced.

## Model specifications and proposals for blades tip leakage flow control

**Figure 1:**
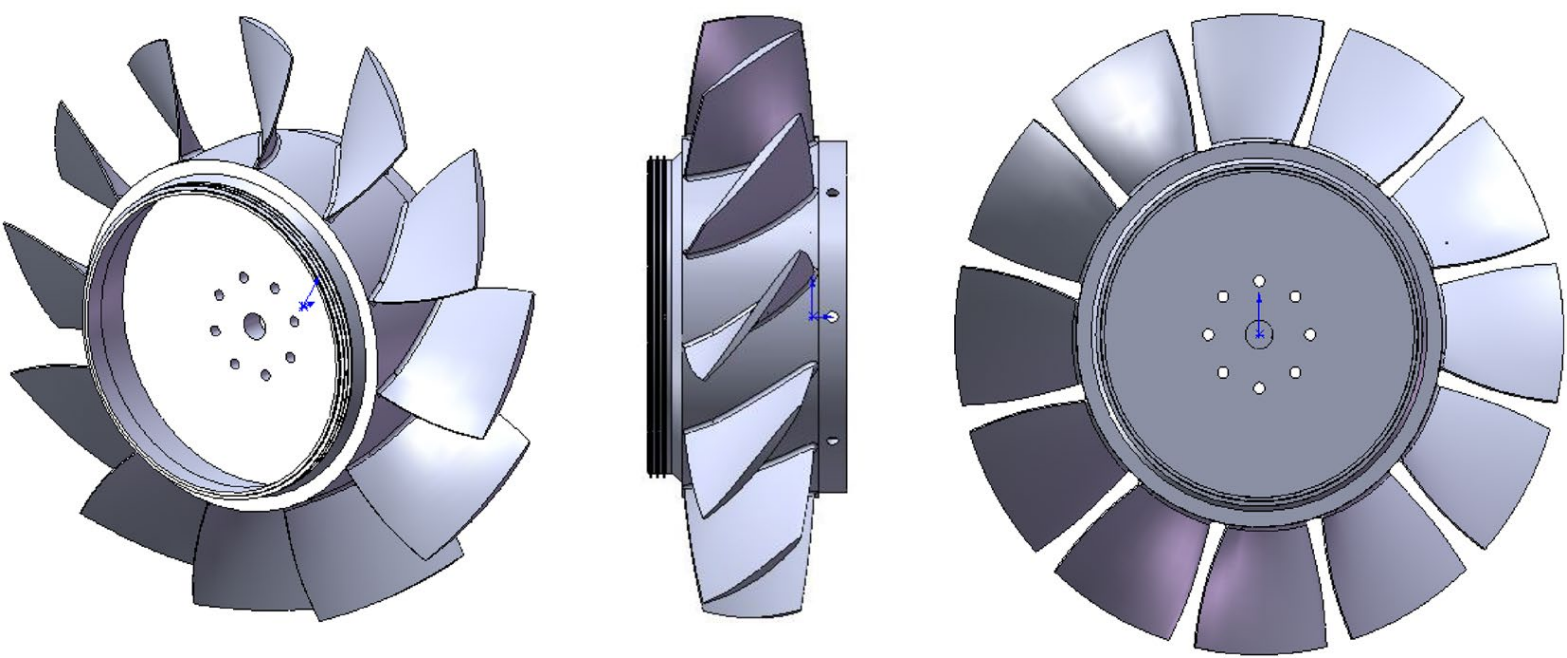
Different views of rotor blades row.

Figure 1 shows different views of the model of investigation, which is a rotor blades row of an axial compressor consisting of 12 blades of NACA-65 series. Rotational speed of this model is 2000 rpm and its geometric specifications is introduced in Table 1. This rotor blades row has already been tested by some researchers like Inoue et al.^10^ and Taghavi Zenouz et al.^11^, experimentally.

**Table 1:** Blades row specifications.

Parameter	Value	Unit
Hub diameter	270	mm
Hub/tip ratio	0.6	–
Tip clearance/blade chord	1.7	%
Tip chord length	117.5	mm
Blades tip solidity	1	–
Blades tip stagger angle	56.2	deg

**Figure 2:**
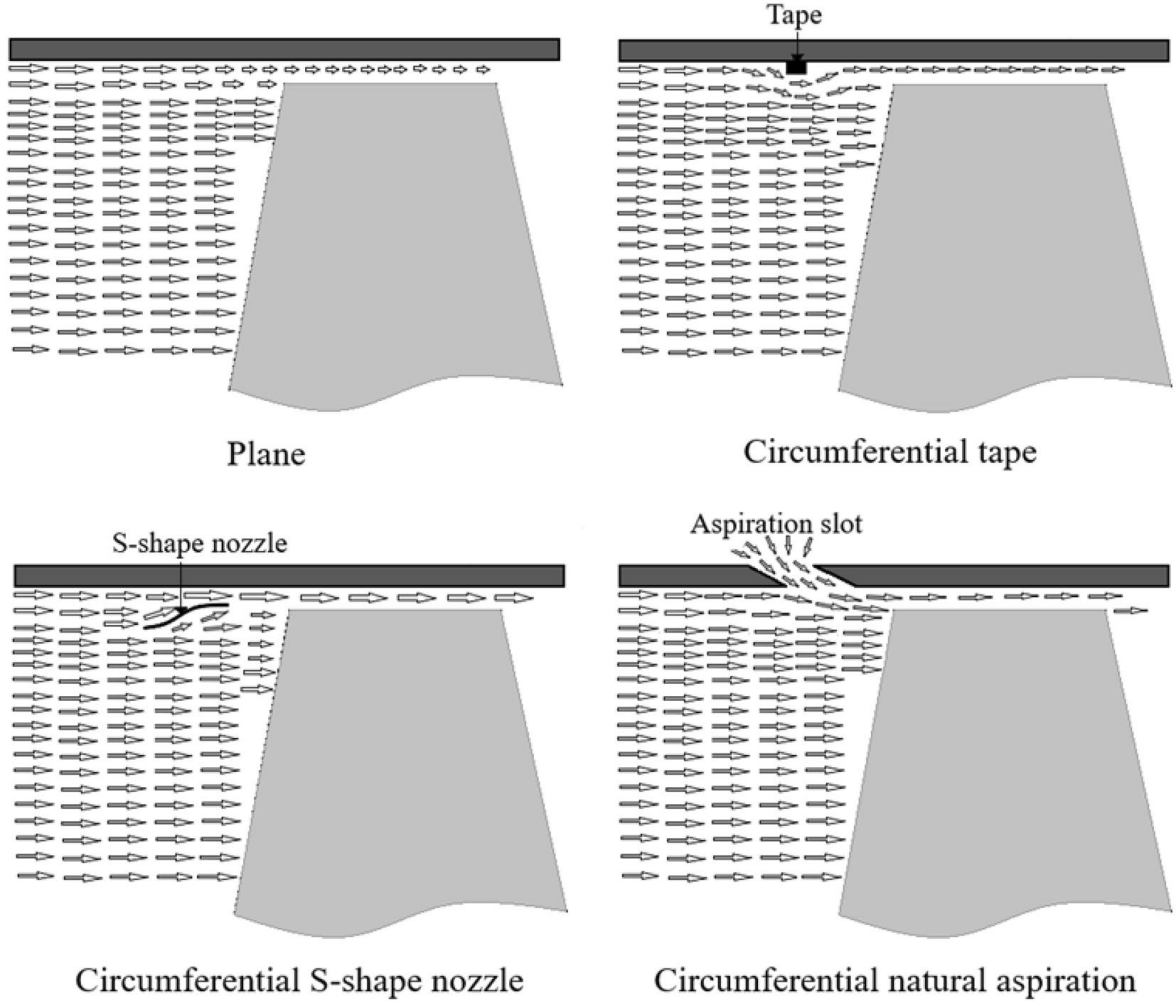
Schematic drawings of different proposals for tip leakage flow control.

Three different methods are proposed to alleviate the undesirable effects of the rotor blades row tip leakage flow. Figure 2 schematically introduces these ideas including the plane case (i.e., without any treatment). These ideas include circumferential tape, S-shape nozzle and natural aspiration, which are all imposed upstream the rotor blades row.

Generally speaking, growth of the boundary layer formed from the engine inlet towards the compressor causes deterioration of flow structure in the blades tip region. Therefore, it would be beneficial to apply techniques for energization of the incoming flow, particularly close to the casing walls. All the three above-mentioned methods are introduced in this respect, with the following specifications for each one.

**Figure 3:**
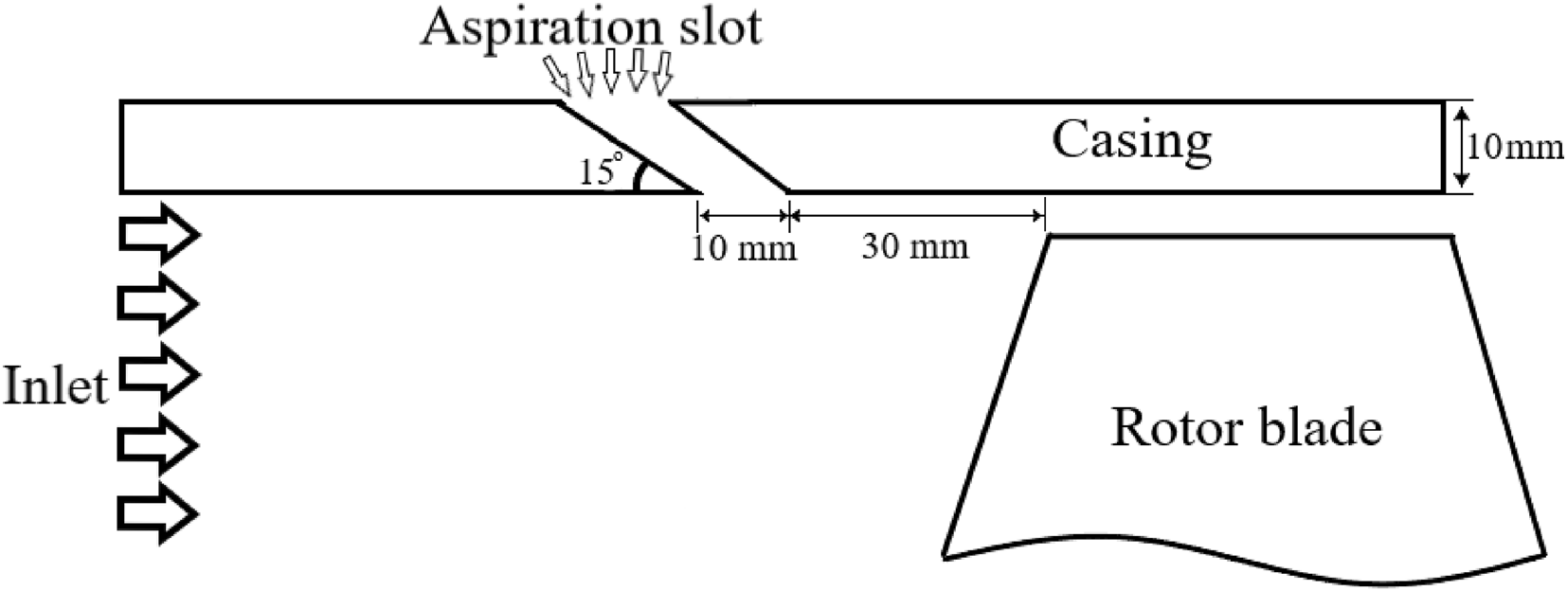
Schematic drawing of meridional view of rotor blade and aspiration slot.

- Circumferential tape with a thickness of 2 mm and 10 mm in length, installed 10 mm upstream the blades row leading edge.
- Circumferential S-shape nozzle with total length of 11.04 mm and inlet and outlet heights of 10 mm and 6 mm, respectively. This nozzle is installed 30 mm upstream the blades row. The equation of this nozzle wall obeys the curvature of the nozzle section of a subsonic wind tunnel designed by Bell and Mehta^12^, which is introduced by Eq. (1)
  1 $$y \left( x \right) = H_{i} - \left( {H_{i} - H_{e} } \right) \left( {6x^{5} - 15x^{4} + 10x^{3} } \right)$$
- Circumferential natural aspiration through a continuous slot implemented upstream the blades within the compressor casing wall. It is inclined 15° relative to the axial direction with a width of 10 mm and is located 30 mm upstream the blades row. Figure 3 shows schematic drawing of this configuration.

## Numerical simulation procedure and boundary conditions

**Figure 4:**
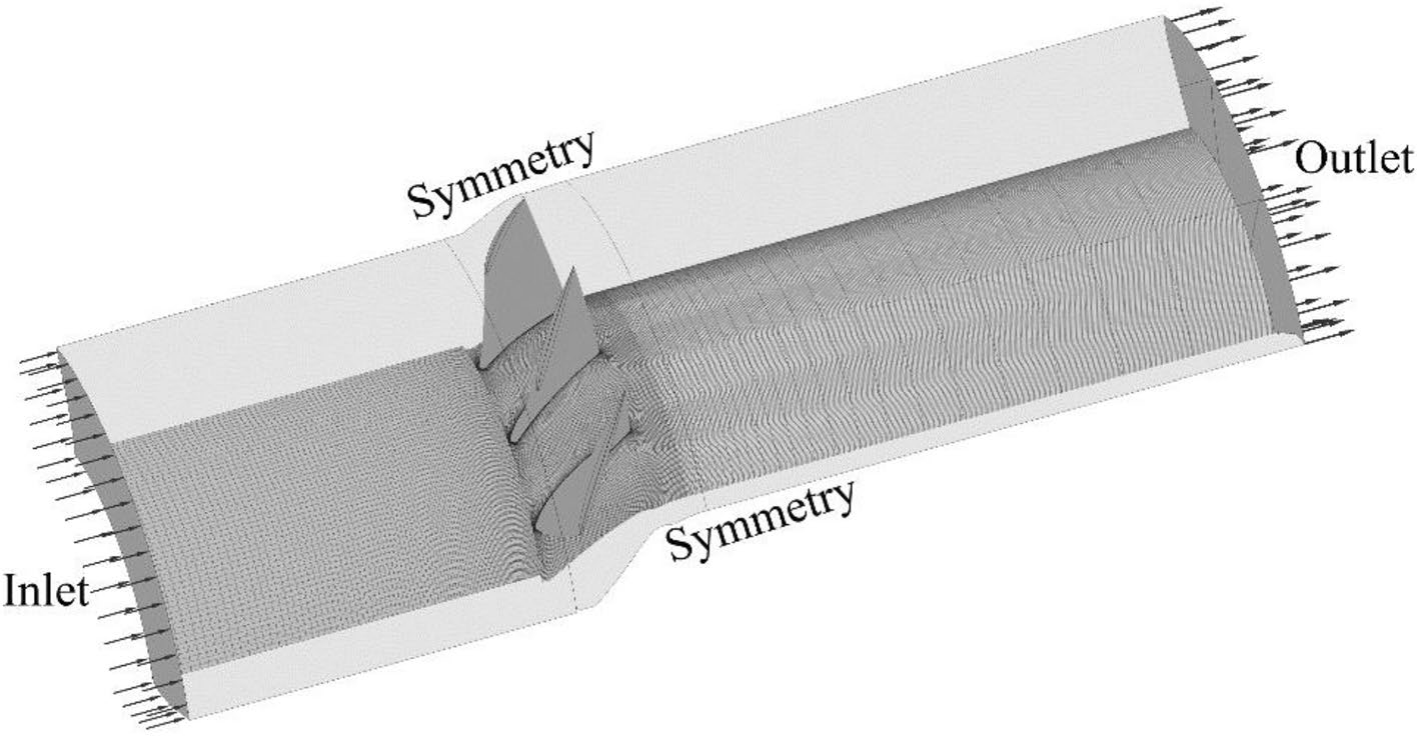
Solution domain and types of boundaries.

As shown in Fig. 4, one quarter of the blades row, including three blades, are considered for the flow simulations. This figure demonstrates the solution domain, which extends three and five times the rotor blade tip chord length upstream and downstream the blades, respectively. The solid walls are considered as adiabatic and periodicity condition is used for the lateral sections. The sliding mesh technique is imposed either side of the rotor blades row. The frozen rotor boundary condition is considered for the interface planes between the stationary and rotating regions.

Table 2 summarizes the boundary conditions used in the present study.

**Table 2:** Boundary conditions.

Inlet boundary	Outlet boundary
Total pressure: 101 kPa	Static pressure: 101–300 kPa
Total temperature: 288.15 K	

The commercial ANSYS-CFX 18.2 is employed for the flow simulation. In addition, ANSYS CFX-Pre is used to define the boundary conditions and ANSYS CFX-Solver, is utilized to solve the governing equations, including the conservation of mass (Eq. 2)^13^, momentum (Eq. 3)^14^ and energy equations (Eq. 4)^15^.

2 $$\frac{1}{\rho }\left( {\frac{D\rho }{{Dt}}} \right) + \nabla \cdot u = 0$$

3 $$\rho \left( {\frac{Du}{{Dt}}} \right) = \rho \left( {\frac{\partial u}{{\partial t}} + u \cdot \nabla u} \right)$$

4 $$\dot{E}_{in} - \dot{E}_{out} = \frac{{dE_{cv} }}{dt}$$

The CFX-Post software is used for the post-processing purposes. The SST k–ω turbulence model is used through the flow simulations process. The k–ω equations provide resolving the viscous sub-layer, precisely. In addition, this model is accurate and trustworthy for flows including adverse pressure gradients in comparison to the other turbulence models. The relevant equations are introduced by the following equations^16^:

5 $$\frac{D\rho k}{{Dt}} = \tau_{ij} \frac{{\partial u_{i} }}{{\partial x_{j} }} - \beta^{*} \rho \omega k + \frac{\partial }{{\partial x_{j} }}\left[ {\left( {\mu + \sigma_{k1} \mu t} \right)\frac{\partial k}{{\partial x_{j} }}} \right]$$

6 $$\frac{D\rho \omega }{{Dt}} = \frac{{\gamma_{1} }}{{v_{t} }}\tau_{ij} \frac{{\partial u_{i} }}{{\partial x_{j} }} - \beta_{1} \rho \omega^{2} + \frac{\partial }{{\partial x_{j} }}\left[ {\left( {\mu + \sigma_{\omega 1} \mu t} \right)\frac{\partial \omega }{{\partial x_{j} }}} \right]$$

In the above equations,$u, x, t,$ $\rho$, and $\tau$ are velocity, distance, time, density, and shear stress, respectively.

### Mesh independency and validation

**Figure 5:**
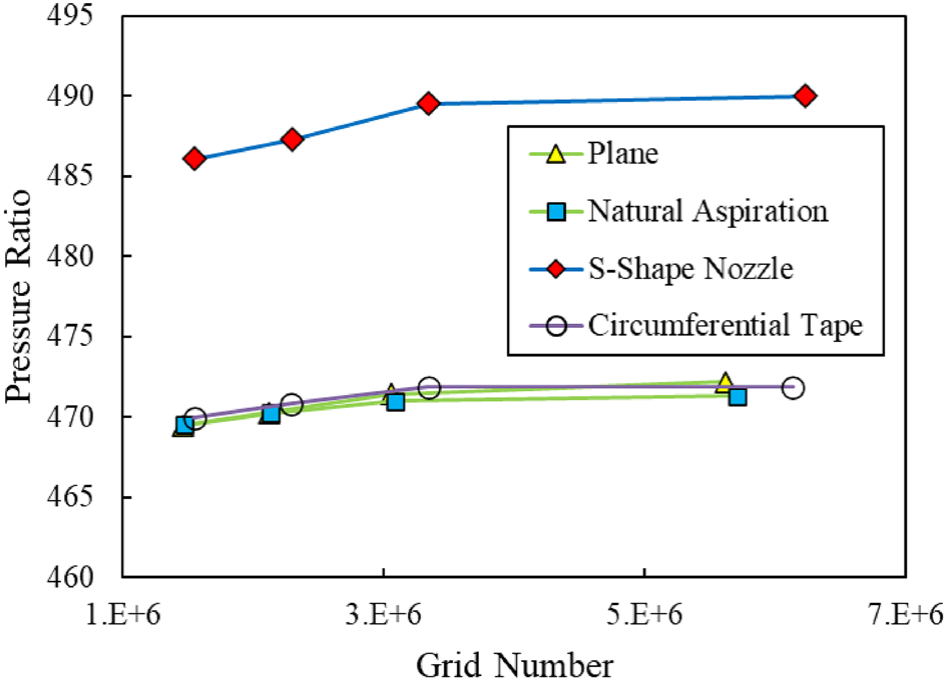
Grid independency results for plane and treated cases.

The average output total pressure versus various number of meshes was calculated for all the four cases, already introduced in section “Model specifications and proposals for blades tip leakage flow control”, and results are shown in Fig. 5. It can be detected from this figure that the pressure does not vary while considering the mesh number greater than about 3 million.

Table 3 introduces the number of meshes for each case. 44 nodes have been radially distributed within the blade tip clearance region for each case.

**Table 3:** Number of meshes for different cases.

Case study	Solution domain
Plane (No Treatment)	3,057,180
Circumferential Tape	3,342,180
Circumferential S-Shape Nozzle	3,345,180
Circumferential Natural Aspiration	3,092,181

**Figure 6:**
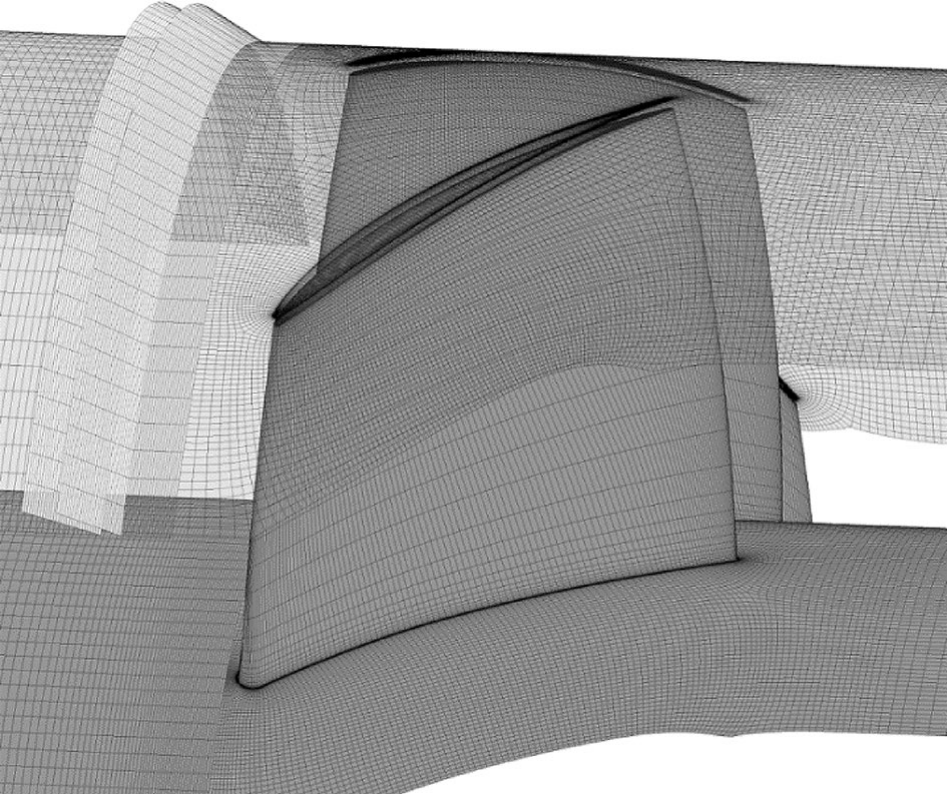
Grid distribution on solid surfaces of circumferential natural aspiration case.

The meshes of structured type, distributed on the solid surfaces of the circumferential natural aspiration case, are shown in Fig. 6.

**Figure 7:**
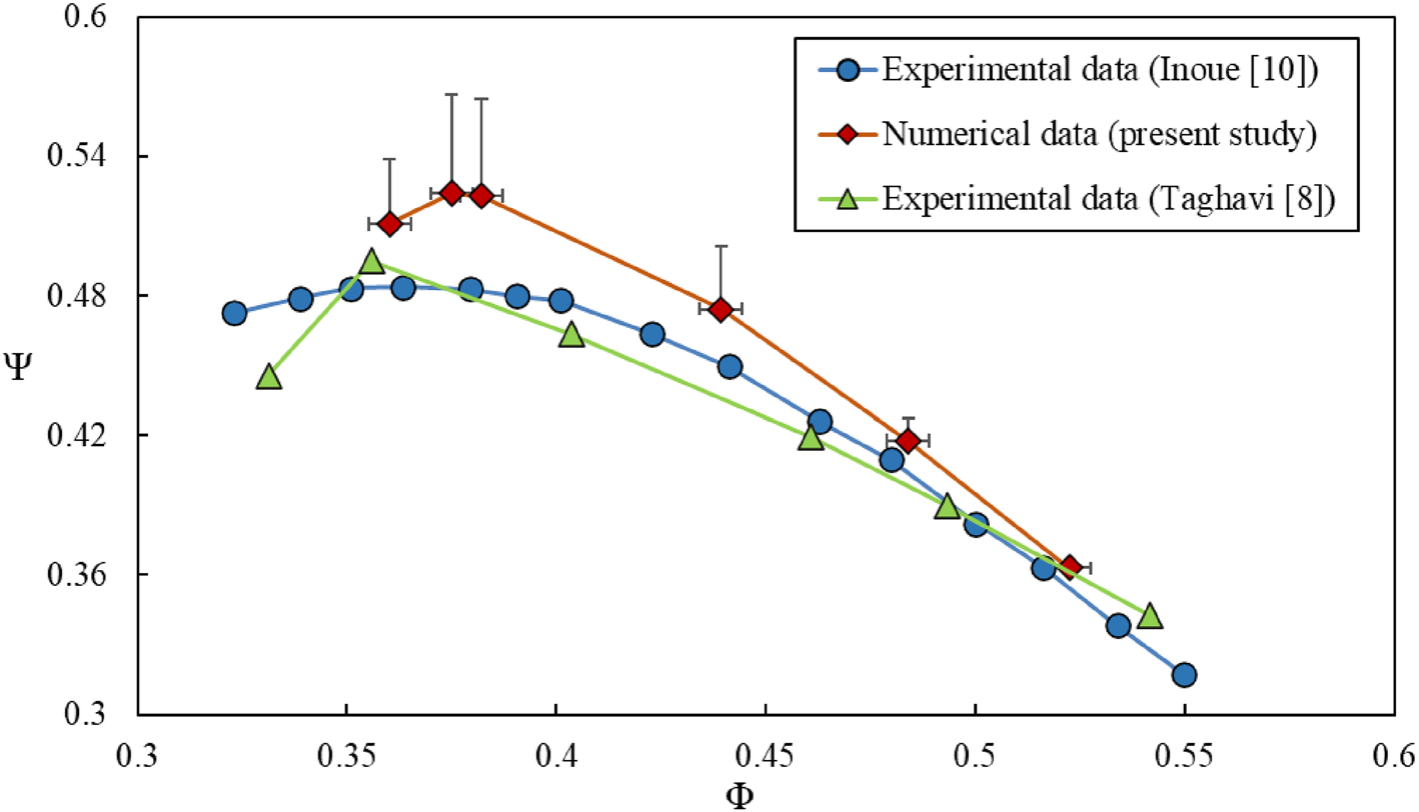
Comparison of performance curves between present results and available experimental data^8,10^.

To validate the numerical results, performance curve of the plane case (i.e., no treatment) in terms of the total pressure rise coefficient $\left( {\psi = \frac{{{\Delta }P}}{{\frac{1}{2}\rho U^{2} }}} \right)$ versus the flow coefficient $\left( {\phi = \frac{{C_{a} }}{U}} \right)$ are compared with those of Inoue et al.^10^ and Taghavi et al.^8^ which are carried out experimentally. These results are shown in Fig. 7. It can be deduced from this figure that the maximum difference between the present numerical results with each of the experimental results is about 12%, which seems appropriate.

## Results and discussion

**Figure 8:**
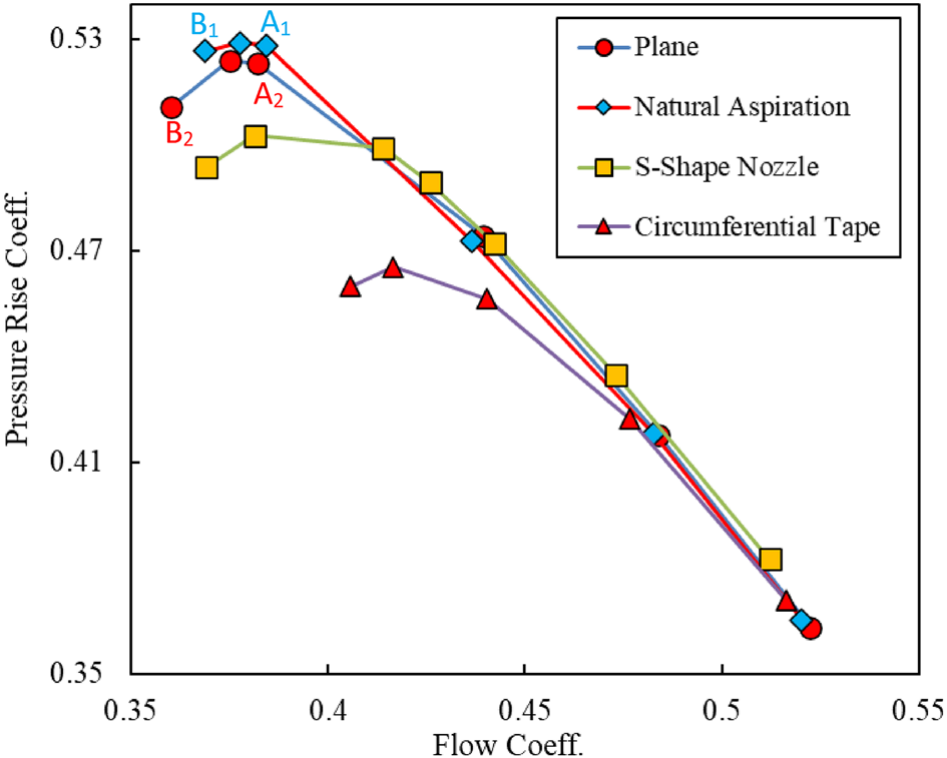
Performance curves for different cases.

Performance curves, in terms of the total pressure rise coefficient versus the flow coefficient, are presented in Fig. 8 for all the case studies already introduced in section “Model specifications and proposals for blades tip leakage flow control”. It can be deduced from this figure that at far stall situations, results coincide each other for $\phi$ > 0.47. With exception to the circumferential tape case, the performance curves of the other cases overlap each other for $\phi$ > 0.4. The worst case belongs to this case, where the stall phenomenon has occurred at very low pressure ratios in comparison to the other cases. Results of the circumferential S-shape nozzle show slight increase of the pressure rise in comparison to the other cases for $\phi$ > 0.4. However, stall has occurred more rapidly in comparison to the plane and natural aspiration cases. Based on the results presented in Fig. 8 the circumferential natural aspiration shows improvement in the performance of the plane case.

**Figure 9:**
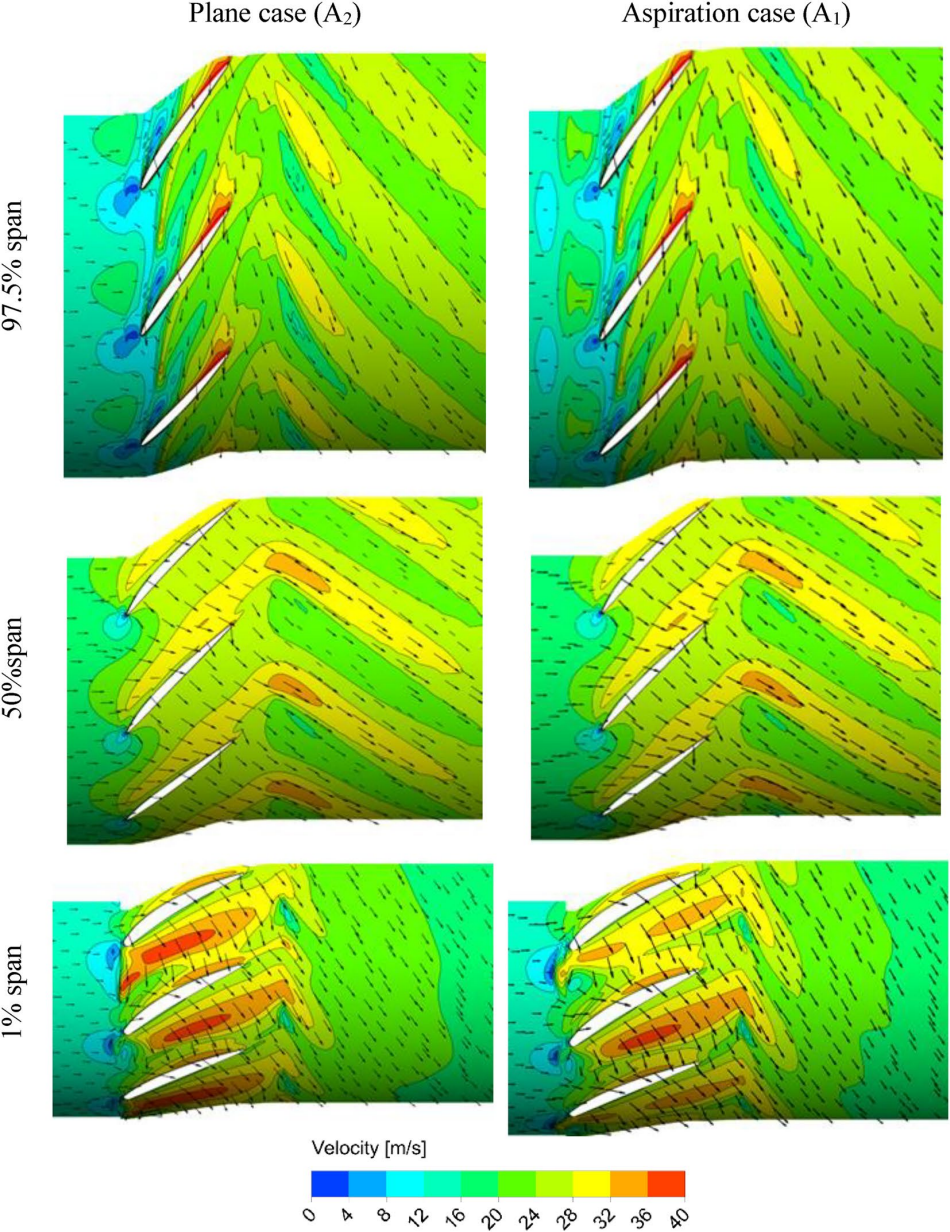
Velocity distribution at different radial positions at pre-stall condition.

**Figure 10:**
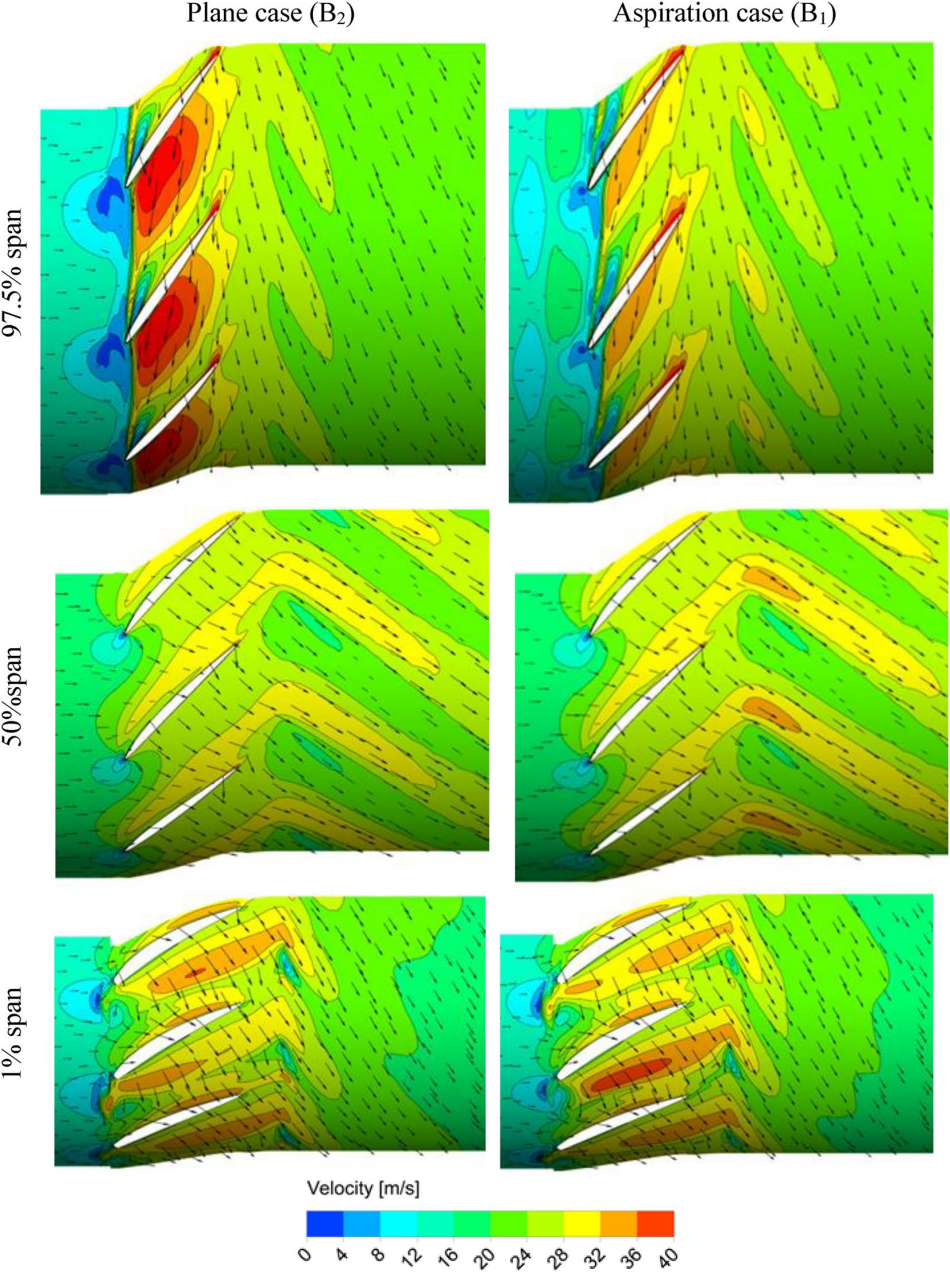
Velocity distribution at different radial positions at post-stall condition.

For better understanding of the governing physics behind the best treatment method, i.e., the natural aspiration case, flow property at vicinity of the stall condition is analyzed, and then, results are compared with those of the plane case. As shown in Fig. 8, two points are specified either side of the maximum pressure rise which are designated by A_1_ and B_1_ for the aspiration case and A_2_ and B_2_ for the plane case. A_1_ and A_2_ refer to the near or pre-stall and B_1_ and B_2_ refer to the post-stall conditions. Results of the flow structure at three spanwise positions, i.e., near the blade root (1% span), mid-span (50% span) and near the tip region (97.5% span) are presented in Figs. 9 and 10 for the near-stall (points A_1_ and A_2_) and post-stall cases (points B_1_ and B_2_), respectively.

As can be detected from Fig. 9, there cannot be seen any significant difference between the results of the plane and aspiration cases for the pre-stall case. Nevertheless, slight increase in the velocity magnitudes can be observed at the entry region of the blades tip row, while using the circumferential natural aspiration. This velocity increment at the blades tip region points out the fact that the main flow is less blocked, in comparison to the plane case. As a result, the blades row can produce a higher pressure rise (compare pressure rise coefficients at points A_1_ and A_2_ in Fig. 8).

Results of post-stall condition, shown in Fig. 10, indicate higher flow velocities at the blades entry region near the tip (97.5% span) in comparison to the plane case. Not a considerable improvement was obtained for the two other radial positions. Similar to the pre-stall case, the entry velocity increment at the blades tip region is accompanied by higher pressure rise (compare pressure rise coefficients at points B_1_ and B_2_ in Fig. 8).

**Figure 11:**
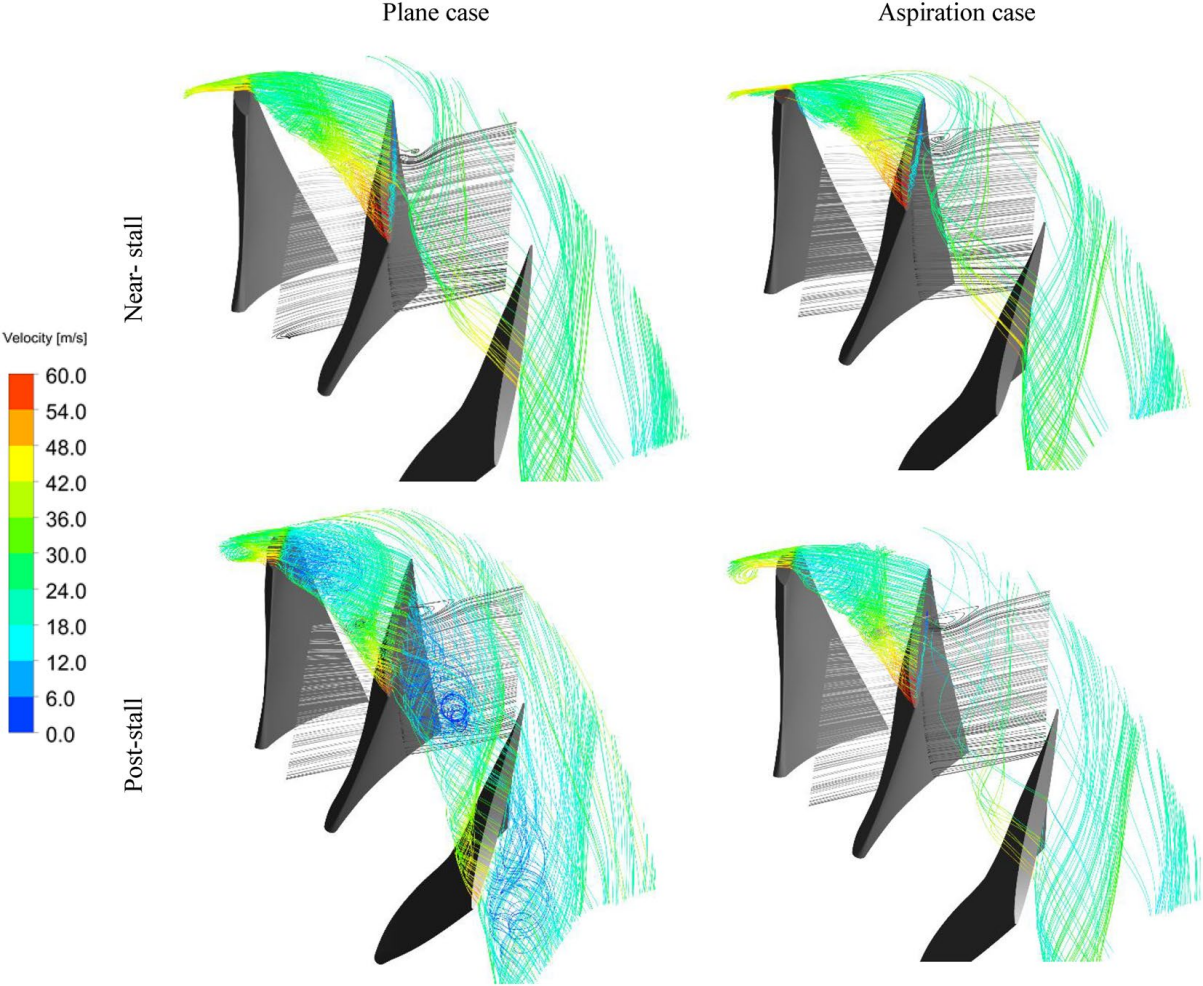
Streamlines at blades row tip region.

Figure 11 shows some streamlines at the blade tip region, extracted from the flow simulations process for the plane and aspiration cases at the near and post-stall conditions. The streamlines are nearly the same for the two cases at the pre-stall condition (points A1 and A2 in Fig. 8). However, beneficial effects of aspiration can be observed for the post-stall condition (points B1 and B2 in Fig. 8). The vortical flows with local low velocities in the post-stall case are diminished in the aspiration case.

**Figure 12:**
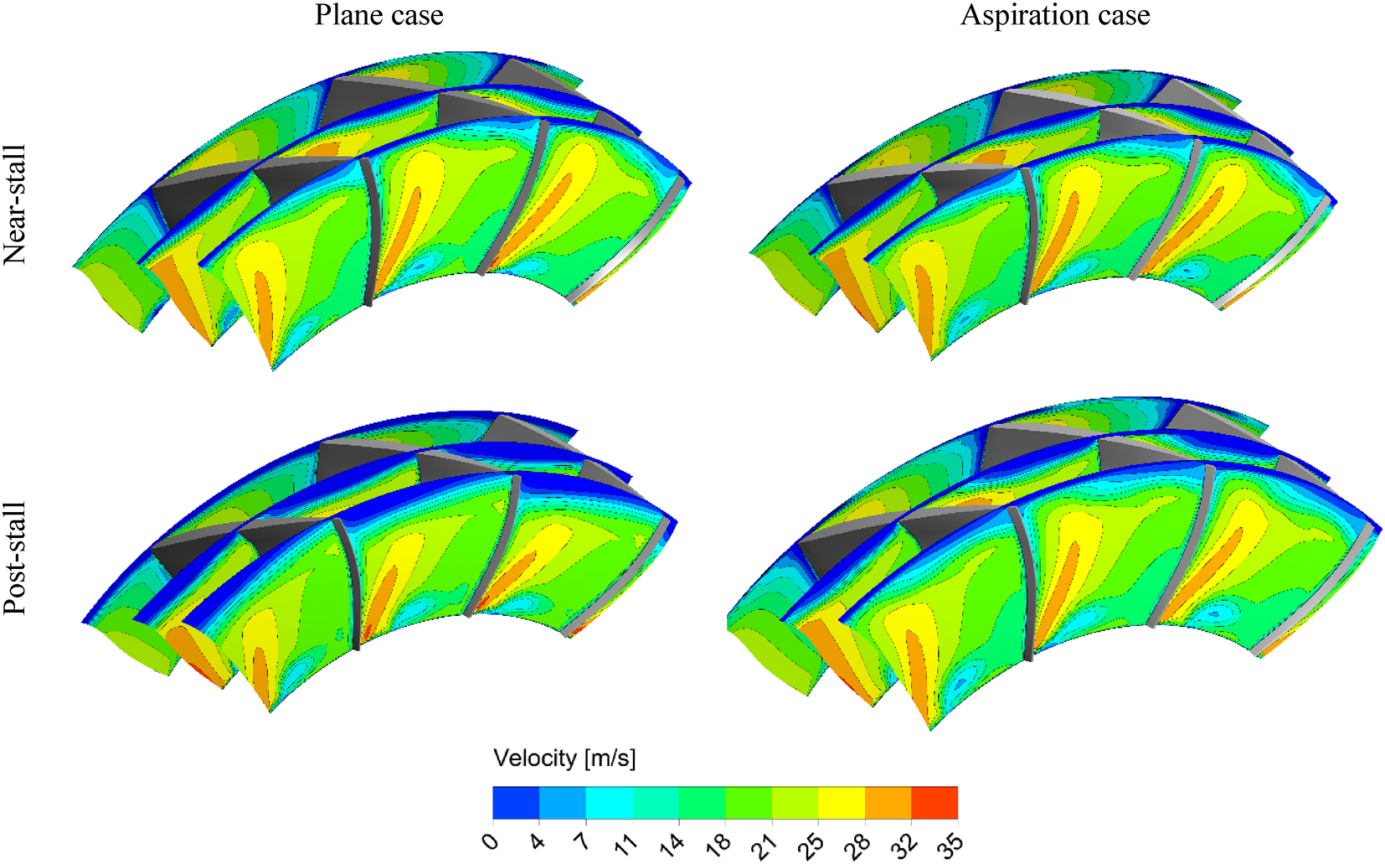
Velocity contours on planes perpendicular to compressor axis.

Figure 12 demonstrates the velocity contours on three planes perpendicular to the compressor axis. Higher velocities at the blades tip region in the natural aspiration case is apparent for the post-stall condition, in comparison to the plane case.

To quantify the beneficial effects of the circumferential natural aspiration, mass flow rates passing through the blades row tip gap for different conditions are calculated and results are presented in Table 4. It can be concluded from this table that the natural aspiration case is accompanied by 3.5% augmentation in the blades tip gap mass flow rate, indicating its beneficial effects.

**Table 4:** Mass flow rate passing through blades row tip gap.

State	Plane case	Natural aspiration case
Near-stall	23.54 gr/s	23.57 gr/s
Post-stall	20.27 gr/s	22.60 gr/s

## Conclusion

Three different passive control methods of circumferential tape, S-shape nozzle and natural aspiration slot are proposed to enhance the tip leakage flow structure in an axial low speed compressor. Following conclusions can be withdrawn from the present research work.

- All three suggested cases of circumferential tape, S-shape nozzle and natural aspiration slot show nearly the same performance as that for plane case for flow coefficients more than 0.47.
- The worst case belongs to circumferential tape case, where stall phenomenon occurs at very low pressure ratios in comparison to other cases.
- Results of circumferential S-shape nozzle show slight increase of total pressure rise in comparison to other cases for flow coefficients greater than 0.4.
- Circumferential natural aspiration case shows improvement in performance of no-treated case. Aspiration causes fluid particles to accelerate at blades entry near their tip region in comparison to plane case at post-stall condition.
- Blades tip vortical flows with local low velocities are being diminished in post-stall condition while using circumferential aspiration.
- Natural aspiration case is accompanied by 3.5% augmentation in blades tip gap mass flow rate at post-stall condition in comparison to plane case.

## Data Availability

The datasets generated and/or analyzed during the current study are not publicly available but are available from the corresponding author on reasonable request.

## References

[1] Lakshminarayana B (1995). Fluid Dynamics and Heat Transfer of Turbomachinery.

[2] Rains, D. A. *Tip Clearance Flows in Axial Flow Compressors and Pumps*. California Inst Of Tech Pasadena Mechanical Engineering Lab (1954).

[3] Storer, J. A., & N. A. Cumpsty. An approximate analysis and prediction method for tip clearance loss in axial compressors. in *Turbo Expo: Power for Land, Sea, and Air*. 78880. American Society of Mechanical Engineers (1993).

[4] Phillips W, Head M (1980). Flow visualization in the tip region of a rotating blade row. Int. J. Mech. Sci..

[5] Zhang, Z., Wu, Y. & Li, Z. Study on unsteady flow characteristic in a transonic axial compressor rotor at near stall condition. in *Turbo Expo: Power for Land, Sea, and Air*. American Society of Mechanical Engineers (2022).

[6] Li J, Hu J, Zhang C (2020). Experimental investigation of the tip leakage flow in a low-speed multistage axial compressor. Sci. Progress.

[7] Agarwal, R. *et al*. Numerical analysis on axial compressor stage performance with vortex generators. *Turbo Expo: Power Land, Sea, Air*. 56635. American Society of Mechanical Engineers (2015).

[8] Taghavi Zenouz R (2017). Experimental investigation on flow unsteadiness during spike stall suppression process in an axial compressor via air injection. Proc. Inst. Mech. Eng. Part G: J. Aerospace Eng..

[9] Hwang, Y. & Kang, S.-H. Numerical study on the effects of casing treatment on unsteadiness of tip leakage flow in an axial compressor. In *Turbo Expo: Power for Land, Sea, and Air*. American Society of Mechanical Engineers (2012).

[10] Inoue M (1991). Detection of a rotating stall precursor in isolated axial flow compressor rotors. J. Turbomach..

[11] Taghavi-Zenouz R, Eshaghi-Sir M, Ababaf-Behbahani MH (2017). Performance of a low speed axial compressor rotor blade row under different inlet distortions. Mech. Sci..

[12] Bell, J. & Mehta, R. *Contraction Design for Small Low-Speed Wind Tunnels, NASA, CR 177488.* Contract NS2-NCC-2-294 (1988).

[13] McLean D (2012). Understanding Aerodynamics: Arguing from the Real Physics.

[14] Babister A (1962). Mechanics of fluids. Aeronaut. J..

[15] Stark J (1966). Fundamentals of Classical Thermodynamics (van wylen, gordon j.; sonntag, richard e.).

[16] Menter, F. Zonal two equation kw turbulence models for aerodynamic flows. In *23rd Fluid Dynamics, Plasmadynamics, and Lasers Conference* (1993).

